# The Important Difference Between Psychologists’ Labs and Real Life: Evaluating the Validity of Models of Wisdom 

**DOI:** 10.1080/1047840X.2020.1750909

**Published:** 2020-06-22

**Authors:** Judith Glück

**Affiliations:** Department of Psychology, University of Klagenfurt, Klagenfurt, Austria

Having studied wisdom for over twenty years now, I think I have learned quite a bit from my own research. If someone describes a difficult life problem to me, I can produce a response that would probably be scored as wise. I consider myself as rather morally grounded, and I have become quite skilled at considering different perspectives, balancing interests, appreciating broader contexts, and knowing the limits of my knowledge. Yet there are moments in my life–family conflicts, endless and useless meetings, interactions with difficult students–where I yell, slam doors, and curse (or at least would like to do so) and where I am neither wise nor act wisely. How is that possible, according to the common model of wisdom proposed by Grossmann et al. (2020)?

In the following, I want to argue that the common model of wisdom is a highly convincing model of wise *reasoning*, especially under laboratory conditions, but may not cover all that is needed for wise *behavior* in real life. In the second part of this paper, I discuss the more general question of how we can test whether a model of wisdom is valid.

First, however, I would like to emphasize that I very much agree with Grossmann et al.’s main point that perspectival metacognitive thinking with a moral grounding is at the core of wisdom. As Igor Grossmann and others have shown in many studies, being aware of the relativity of one’s own perspectives and beliefs and being motivated to achieve some common good for a larger group–be it one’s family, an institution, or a whole nation–are undisputable and central aspects of wisdom (see also Sternberg, 1998, 2019). The model proposed by Grossmann and colleagues is an excellent descriptive model of the commonalities of wise solutions to a range of wisdom-requiring problems. An explanatory model that also accounts for *how* individuals arrive at such solutions would seem to be an important next step, and as I am going to argue in the following, such a model would need to include emotional aspects.

## Wisdom in Real Life vs. Wisdom in the Lab

When and where do we see wisdom “in action” in real life? I would argue that there are two types of situations in which wisdom most typically manifests itself. The first situation is being faced with a difficult situation in one’s own life, such as a serious conflict, a difficult decision, or a highly negative event. In our study of people’s autobiographical wisdom memories (Bluck & Glück, 2004; Glück, Bluck, Baron, & McAdams, 2005), for example, people talked about divorce, mobbing at the workplace, or the sudden loss of a loved one. In such situations, perspectival metacognition and moral grounding are definitely necessary, but I do not think they are quite sufficient for wisdom. Grossmann and Kross (2014) showed that people are significantly less wise when they imagine a challenging situation concerning themselves than when they imagine it concerning someone else–and it seems quite plausible that they would be even less wise if the situation were not imaginary but real. Staudinger (2019) distinguishes between personal and general wisdom, arguing that dealing wisely with difficult life problems of one’s own requires different capacities from dealing wisely with an imaginary stranger’s problem. What exactly is the difference? Certainly, much of it is emotional and self-related. Even if we only imagine our partners cheating on us (the situation that Grossmann and Kross [2014] used in their study), we experience feelings of betrayal, shame, anger, and sadness, and these feelings are much stronger, of course, if such a situation happens to us in real life. Dealing wisely with these feelings would require acknowledging and accepting them, but also being able to regulate them to an extent that enables us to make good decisions and to hurt other people as little as possible in the process. Moral grounding and perspectival metacognition are unlikely to be sufficient for this.

The second prototypical wisdom situation is interactional (Glück et al., 2005; Montgomery, Barber, & McKee, 2002; Yang, 2008). For many people, the most typical manifestation of wisdom is providing support, guidance, and (sometimes) advice to people facing difficult situations like the ones described earlier. A wise individual would be able to support a man who just found out that his wife has been cheating on him, or a woman who is mobbed by her colleagues, in ways that make them resolve the situation, feel better, and perhaps learn and grow from the experience. Doing so certainly requires moral grounding and metacognitive perspective-taking, but, again, I think it also requires emotional competencies: the ability to empathize with other people even if they are very different from oneself, but not to empathize so much that one cannot also see the larger picture. By being tuned in to emotions and situational contexts, but also able to take a broader perspective, wise individuals can open up new perspectives and insights to other people. In sum, I believe emotional and self-regulatory competencies may not play a major role for responding to theoretical life problems, but they are an important part of wise behavior in real life.

Several existing models of wisdom include components that pertain to these emotional aspects of wise behavior. Ardelt’s three-dimensional wisdom model (Ardelt, 2003; Ardelt, Pridgen, & Nutter-Pridgen 2019) includes compassion as one of its three dimensions. Aldwin, Igarashi, and Levenson (2019; see also Levenson, Jennings, Aldwin, & Shiraishi, 2005) argue that self-transcendence, an experience of interconnectedness and peace of mind that enables individuals to unselfishly care about others and the world at large, is at the core of wisdom. Webster’s (2003, 2007) model includes emotion regulation and humor as components of wisdom gained through reflection on life experiences. Our own developmental model of wisdom proposes that emotional sensitivity and emotion regulation are among the most important resources that enable individuals to gain wisdom-related insights about themselves and life in general (Glück, Bluck, & Weststrate, 2019). I believe that a comprehensive model of wise behavior should include emotion-related components.

Even for a model that limits itself to wise reasoning, however, emotional aspects may play an important role as they influence individuals’ capacity for self-reflection. Perspectival metacognition, as defined by Grossmann and colleagues, includes various reasoning strategies (consideration of different perspectives, epistemic humility, consideration of diverse perspectives, recognizing and balancing different interests) that are crucial for finding a wise solution to a difficult situation. These strategies require individuals to question their own views and beliefs, which, again, can be difficult in challenging situations. To wisely support a close friend in a painful divorce, for example, I need to be highly attentive to my own emotions and wary of the effects they may have on my behavior. I need to carefully regulate my positive and negative feelings toward everyone involved, to be aware of how my reactions to a situation are shaped by my own experience, and to try to empathize with all people involved. In other words, I need to integrate cognition and emotion (Labouvie-Vief, 1990)–to cognitively reflect on and regulate my own and others’ emotional experiences.

In analyzing narratives of difficult autobiographical experiences, Nic Weststrate and I identified two modes of reflection (Weststrate & Glück, 2017a). Redemptive reflection (McAdams, Reynolds, Lewis, Patten, and Bowman, 2001) aims at a sense of emotional closure by transforming an initially negative experience into a positive one (“all’s well that ends well”). Exploratory reflection (Lodi-Smith, Geise, Roberts, & Robins, 2009; Pals, 2006) focuses on insight and growth–on understanding what happened, why it happened, and what one can learn from the experience. In our data, redemptive reflection was correlated to well-being, whereas exploratory reflection was correlated to wisdom (Weststrate & Glück, 2017a). Why do many people use redemptive rather than exploratory reflection? Again, the reason is probably emotional: exploratory reflection can hurt. Growing wiser from a conflict with my teenage daughter requires me to think deeply about how I contributed to the escalation of an argument, why I get so angry when she says certain things, how my perception of her as a person may be biased by my dreams for her, and so on. These considerations might be painful, as they may threaten a highly identity-relevant aspect of my self-concept–my belief that I am a good mother. It is certainly easier to just blame the conflict on puberty! Grossmann et al. (2020) include “seeing through illusion and self-deception” as a function of perspectival metacognition in their Figure 3. I would argue, again, that seeing through illusion and self-deception requires emotional awareness, emotion regulation, and empathy (Kunzmann & Glück, 2019)–components that are not discussed by Grossmann et al. (2020). (Interestingly, in their Figure 1-B shows equanimity as a major quality of wisdom when it comes to life challenges, but not as an important component of researchers’ working definitions of wisdom.)

One could argue that all these emotional components are already part of the model: that compassion is part of moral grounding and that emotion regulation and self-reflection are part of perspectival metacognition. On page 8, for example, the authors say that moral grounding includes “general moral attributes (i.e., prosocial orientation), as well as specific goals and tendencies (e.g., compassionate attitude, sympathy).” Metacognitive categories include consideration of different perspectives, search for balance between divergent interests, appreciation of the broader context of a given issue, and epistemic/intellectual humility, but at later points, insight and mentalization (a complex construct in itself) also seem to be included. Thus, it may well be that emotional aspects are somehow hidden or implicitly included in the model’s components, but that would make the meaning of the components very broad and complex and their labels not quite adequate. Clear and comprehensive definitions of each component of the model would be very helpful.

My impression is that the model essentially describes characteristics of *wise reasoning*. These aspects are visible from the outside as they manifest themselves in wise solutions to complex problems. The internal emotional and self-related processes that are required to arrive at such solutions in real life are not part of the model. As such, this is perfectly acceptable, but it might be better to call it a model of wise reasoning, just as Baltes and colleagues stated that their model described wisdom-related knowledge, not wisdom per se (Baltes & Kunzmann, 2004, p. 294).

Wise reasoning, like wisdom-related knowledge, has the psychometric advantage that it can be measured by means of theoretical life problems. Laboratory measures that ask participants to think about a fictitious person’s fictitious problem capture participants’ capacity for wise reasoning, but not necessarily their capacity for wise behavior in real life. In fact, this is one reason why several researchers, including myself (Glück, 2018) as well as Grossmann’s group (Brienza, Kung, Santos, Bobocel, & Grossmann, 2018), have called for moving wisdom measures closer to real life–for example, by looking at autobiographical experiences. How do wise individuals manage to utilize morally grounded perspectival meta-cognition in the midst of a conflict with a family member or an obnoxious colleague? I personally believe that it is more interesting, and perhaps more helpful for fostering the manifestation of wisdom in the world, to include these aspects in a model of wisdom than to focus on cognitive aspects. But this is just my perspective and it is certainly influenced by my own experiences, preferences, and biases. In fact, it seems to me that different conceptions of wisdom reflect, to some extent, the personalities of their originators. How can we try to establish the validity of models of wisdom beyond our personal inclinations?

## Establishing the Validity of a Model of Wisdom

How can we prove that a model of wisdom is valid–that it is a “good” and comprehensive representation of the construct in question? To some extent, this question concerns all psychological constructs–how can we prove the validity of a model of intelligence or of extraversion? But somehow the problem seems particularly tricky with wisdom, perhaps because wisdom is such a complex construct: we can design problems that require intelligence and define what an intelligent solution is; we can define how an extraverted person feels and acts in certain contexts. But with wisdom, it feels like we would need a lot of wisdom ourselves to know what a wise solution to a truly complex problem is and how a wise person creates that solution. In the following, I propose four approaches to establish the validity of a wisdom model: evaluating the model’s consistency with people’s views of real-life wisdom, evaluating its consistency with experts’ conceptions of wisdom, empirically testing the consistency of a model with theory-based predictions, and borrowing thought experiments from philosophers. Of course, psychological models will always be constructions–no model of a psychological concept has ever been perfectly “true.” But at least *striving* for as much truth as possible is arguably characteristic of both wisdom and good science (Ardelt, 2003; Glück, 2017).

### Reality Check: Looking at Non-Experts’ Views of Real-life Manifestations of Wisdom

As I said earlier, I believe that our conceptualizations of wisdom should describe wise behavior in real-life situations. No research has actually put people into actual difficult situations to assess their wisdom, but there are quite a few studies that looked at the experiences of so-called laypeople–people who are not experts on wisdom–with wisdom in real life. Some studies asked participants about situations in which they did something wise (Bluck & Glück, 2004; Glück et al., 2005; König & Glück, 2012); other studies asked participants about situations in which someone else did something wise (König & Glück, 2012; Montgomery et al., 2002; Yang, 2008). One consistent finding of these studies is that people typically talk about difficult, uncertain, and emotionally challenging situations–we do not need wisdom to decide whether we want go jogging today; we need it when we hear about a parent’s cancer diagnosis, find out that our spouse wants a divorce, or support a friend in a serious conflict with an abusive boss. These findings seem to support my notion that wisdom involves dealing with strong feelings.

There is also a considerable body of research on people’s conceptions of wisdom in general (overview in Weststrate, Bluck, & Glück, 2019). Starting from the very first study of people’s conceptions of wisdom (Clayton & Birren, 1980), these studies show wisdom conceptions typically include cognitive, reflective, and affective aspects. People frequently associate capacities such as compassion, emotion regulation, or self-transcendence with wisdom.

One could argue that the views of non-experts are not very relevant–after all, they might have it all wrong because they do not know enough about psychology. This may well be true for more specialized psychological constructs such as, say, theory of mind or value relativism, but the concept of wisdom is frequently used in the general public and part of our culturally transmitted knowledge (e.g., Asadi, Khorshidi, & Glück, 2019; Glück, Bischof, & Siebenhüner, 2012; Weststrate, Ferrari, & Ardelt, 2016). While a scientific model of wisdom can certainly be more elaborate and sophisticated than the typical conceptions of non-experts, it would be difficult to defend a model that is seriously incompatible with them.

An interesting way to explore the validity of different models of wisdom might be to test their compatibility with people’s views more directly than has been done before. For example, researchers could construct vignettes describing how a protagonist deals with a difficult situation, systematically varying characteristics of the situation and the protagonist’s behavior. Participants would then be asked to rate the wisdom of the protagonist. Would they rate his or her behavior as equally wise if, for example, the same behavior is described as the result of “cold” cognition as when it is described as the result of compassionate concern? Such research could enrich our knowledge about which characteristics people consider as necessary and/or sufficient for wisdom.

### Standing on the Shoulders of Others to Get a Broader View

It is a great idea to collect the views of experts on what wisdom is. If the experts in the field agree on a model, that should be a very convincing indicator of its validity. The most common approach to collect experts’ views is to look at published definitions of wisdom–after all, they are carefully crafted representations of the thoughts of their creators (for overviews see, e.g., Glück, 2015; Staudinger & Glück, 2011). I was a bit surprised that Grossmann et al. (2020) did not review existing models of wisdom: the Berlin wisdom model (e.g., Baltes & Staudinger, 2000), Ardelt’s three-dimensional model (e.g., Ardelt, 2003), or Sternberg’s balance theory (e.g., Sternberg, 1998, 2019) are mentioned in passing, but the definitions and components of wisdom in those models are not described. In fact, to readers unfamiliar with the field, it may seem as if no one had proposed a comprehensive model of wisdom before. I was particularly surprised that Sternberg’s balance theory of wisdom (e.g., Sternberg, 1998, 2019) is not given much notice, as it essentially defines wisdom as the morally-grounded ability to balance different interests, which is quite similar to the new common model. (For readers who would like to read more about the whole range of current wisdom research, the new Cambridge Handbook of Wisdom [Sternberg & Glück, 2019a] may be a good starting point.)

Rather than review published models of wisdom, the authors report the results of a survey of wisdom researchers’ conceptions of wisdom. This approach certainly has the advantage of including a larger group of experts, people who may have thought about wisdom a lot but not, or not yet, published any conceptions of their own. To date, two expert surveys on components of wisdom have been published. One is the current paper by Grossmann and colleagues. The other was published by Jeste, Ardelt, Blazer, Kraemer, Vaillant, and Meeks in *The Gerontologist* in 2010. In that study, 30 wisdom experts rated the importance of 47 characteristics with respect to wisdom on a scale from 1 (definitely not important) to 9 (definitely important). In descending order, the characteristics that had average ratings of 8 or higher were “Recognizing limits of one’s own knowledge” (8.8), “maturity gained with experience” (8.6), “self-reflection” (8.6), “self-insight” (8.6), “tolerance of differences among others” (8.5), “rich knowledge of life” (8.4), “social cognition” (8.4), “acceptance of uncertainty in life” (8.4), “sense of justice or fairness” (8.4), “empathy” (8.3), “tolerance of ambivalence” (8.3), “value relativism” (8.2), “openness to new experience” (8.2), “learning from experience” (8.2), “ability to give good advice” (8.2), “ethical conduct” (8.2), “practical life skills” (8.1), “emotional regulation” (8.0), and “desire for learning/knowledge” (8.0). In sum, there is much overlap between Jeste et al.’s (2010) and Grossmann et al.’s (2020) findings–most of the aspects from Jeste et al.’s (2010) survey can be categorized into either “perspectival meta-cognition” or “moral grounding.” But there is also at least one major difference, and it is the same one I keep coming back to. Aspects that have anything to do with empathy or emotion regulation are not part of the common model.

One reason for the model’s focus on wise reasoning may be that it is not just a common model but a common-denominator model: it focuses on those components of wisdom on which all or most wisdom researchers in the survey agreed. There obviously was less consensus on emotion-related aspects of wisdom, because wisdom researchers come from different backgrounds and focus on different aspects of this complex construct. (As an aside, it would be very interesting to know more about the representativeness of the sample across different “schools” of wisdom research, but this information is difficult to collect in an anonymous sample). It seems unlikely to me, however, that all of wisdom is really captured in those aspects that everyone agrees on. In “The Wisdom of Crowds”, Surowiecki (2005) argues that a group can be smarter than the smartest of its members if (a) the members of the group are sufficiently heterogeneous in background, and (b) the group values and encourages this heterogeneity and takes every voice seriously. A common-denominator approach does not seem to do that.

### Testing Empirical Predictions

In addition to considering the views of experts and non-experts, a model of wisdom can, to some extent, be tested empirically. For example, Ardelt (2004) argued, in her critique of the Berlin wisdom model, that a measure of wisdom ought to show a certain relationship with age. If a measure shows a relationship with age that is highly inconsistent with what one would expect for wisdom, such as general decline in old age, the measure and, in turn, the model of wisdom it is built on, may not capture the essence of wisdom. Along the same lines, one can make predictions about the relationship between wisdom and other variables. To some extent, this argument is circular because our ideas about the relationship between wisdom and other constructs are, again, influenced both by people’s general conceptions and by our own theories of wisdom, but at least a certain extent of plausibility should be deducible from such approaches.

What relationship would we predict for wisdom and age? Neither non-experts (Glück & Bluck, 2011) nor experts (e.g., Ardelt, 2004; Glück, 2019; Jeste et al., 2010; Staudinger, 1999) believe that wisdom increases linearly with age–that is, that everyone grows wiser with age. The path to wisdom is rocky and steep and requires critical self-examination (Weststrate & Glück, 2017b), which not everyone wants to engage in. The most likely prediction for the relationship of wisdom and age is that there would be no relationship for most people, but a small subgroup of people should reach their highest levels of wisdom in early old age (Glück, 2019; Staudinger, 1999). I have yet to see a measure of wisdom that shows this pattern.

Generally, I agree with Ardelt (2004) that we need to distinguish between research results obtained with well-validated measures of wisdom and research results with measures of wisdom that may not even really be measures of wisdom. We first need to establish, as convincingly as possible, that a measure is indeed assessing wisdom before we discuss the substantive implications of our findings with that measure. For now, I have most faith in findings that are consistent across different measures of wisdom (e.g., Glück, Gussnig, & Schrottenbacher, 2019; Weststrate & Glück, 2017a; Webster, Weststrate, Ferrari, Munroe, & Pierce, 2018). Based on this approach, to briefly come back to my first point, there actually is empirical evidence that wisdom is at least related to aspects of compassion, emotional sensitivity, emotion regulation, and self-reflection. Testing our model of the development of wisdom, we found reliable relationships between these variables and several different measures of wisdom (Glück et al., 2019).

### Borrowing from Philosophers: Thought Experiments

I learned a lot from teaching a course on wisdom together with a philosopher–a lot about wisdom, but also a lot about the different ways different disciplines go about understanding a concept (and a lot about how researchers from different disciplines can just look at each other in disbelief: how can you think this question is important and that question is not?). One thing l learned is that we might gain a lot of clarity and precision in our understanding of wisdom if we borrow certain thought experiments from philosophy. For example, if we want to decide whether quality X is a component of wisdom, we could ask ourselves questions such as, (1) Can a person be wise who does not have quality X?, (2) Can a person have quality X and not be wise?, or (3) If we were to boost quality X by some kind of intervention, would that intervention also boost wisdom? For example: (1) Can a person be wise who does not have moral grounding? (2) Can a person have moral grounding without being wise? (3) If we were to boost a person’s moral grounding, would we automatically also increase that person’s wisdom? My answers to these questions would be no, yes, and probably yes. I fully agree with Grossmann and colleagues and other researchers such as Sternberg (1998, 2019; Sternberg & Glück, 2019b) that a wise person necessarily has a strong moral grounding. A person who has moral grounding is not necessarily wise, because the combination with other qualities, including the capacity for perspectival meta-cognition, is necessary for wisdom. Making a person more morally grounded would probably make him or her wiser, but there might be conditions (such as a lack of cognitive complexity) that might prevent the person from reaching high levels of wisdom.

Thinking about components of wisdom theoretically in this way, and especially discussing such questions in interdisciplinary teams of psychologists and philosophers, might enable us to arrive at more precise models of which qualities are truly necessary components of wisdom and which qualities are either predecessors that foster the development or manifestation of wisdom or outcomes of some components of wisdom. For example, wise individuals show more gratitude than other people do (König & Glück, 2013, 2014), but I would not consider gratitude a component of wisdom: even if wise individuals are typically grateful, there are lots of highly grateful people who are not particularly wise, and boosting gratitude is unlikely to also boost wisdom.

To sharpen the profile of wisdom in this way, I suggest we look into closely related concepts. Intelligence may be too distant to really render important insights into wisdom–most wisdom researchers and laypeople probably agree that a certain amount of intelligence is necessary for wisdom, but beyond that, it does not matter a lot (Glück, 2020; Webster, 2010). But can a person, for example, be highly forgiving (Taylor, Bates, & Webster, 2011) but still very unwise? Can a person be too compassionate or too self-critical to be wise? Can a person’s behavior be perfectly moral and still unwise? It might also be interesting to look more closely at *non*-wisdom: are there certain characteristics that immediately render a person or a behavior unwise (in the eyes of wisdom researchers and/or non-experts), even if other aspects of wisdom are present? Could, for example, a decision that is based on a gut feeling be considered as wise if it is morally sound and balances different interests, even if it has not been made by explicit wise reasoning? Such considerations could help us both to sharpen our understanding of wisdom and to derive new hypotheses for empirical studies.

## What is a Common Model Good For?

I would like to end this commentary by asking what the advantages of a common model of wisdom in a strong sense–a model that all researchers agree on and that we use in a subsequent “organized effort” to understand wisdom better–would be. When I entered wisdom research in 1999, we essentially had a common model: the Berlin wisdom model and its measurement paradigm strongly dominated wisdom research. The Berlin group’s research was of tremendous importance for getting empirical wisdom research started, and I still consider it as one of the most convincing approaches to studying wisdom. However, I remember how much richer and more colorful wisdom research became as it grew both in depth and in breadth when other conceptualizations and empirical approaches were published. We would not be where we are today if we had exclusively relied on the Berlin model. Sure, empirical findings on wisdom diverge, sometimes strongly, according to which measure of wisdom is used (e.g., Glück, 2019), but I am not sure what we really gain if we all focus on one particular approach. Would intelligence research profit from a collective decision for one intelligence model and measure? Did personality research as a whole really profit from the long-term focus on the Big Five? As an alternative, we have suggested using multimethod approaches and explicitly analyzing the differences in results between different measures of wisdom, in order to understand how study results depend on the underlying wisdom conceptions (e.g., Glück, Gussnig, & Schrottenbacher, 2019; Weststrate & Glück, 2017a; Webster, Weststrate, Ferrari, Munroe, & Pierce, 2018). As mentioned earlier, the wisdom of crowds manifests itself in the appreciation of many different voices (Surowiecki, 2005). As wisdom researchers, I think we should do just that.
